# Efficacy of Curcumin on Treating Cancer Anorexia-Cachexia Syndrome in Locally or Advanced Head and Neck Cancer: A Double-Blind, Placebo-Controlled Randomised Phase IIa Trial (CurChexia)

**DOI:** 10.1155/2022/5425619

**Published:** 2022-06-02

**Authors:** Tawasapon Thambamroong, Kasan Seetalarom, Siriwimon Saichaemchan, Yanisa Pumsutas, Naiyarat Prasongsook

**Affiliations:** ^1^Division of Medical Oncology, Department of Medicine, Phramongkutklao Hospital and College of Medicine, Bangkok, Thailand; ^2^Division of Clinical Nutrition, Department of Medicine, Phramongkutklao Hospital and College of Medicine, Bangkok, Thailand

## Abstract

**Background:**

Cancer anorexia-cachexia syndrome (CAS) is a significant comorbidity among all patients with cancer, increasing the mortality rate. Almost all patients with head and neck cancer experience this syndrome. CAS causes increased energy expenditure by increasing systemic inflammation and decreasing energy consumption due to anorexia. It leads to skeleton muscle breakdown and reduces the quality of life. Nutritional interventions and primary cancer treatment are the mainstays to manage this situation. However, a vicious cycle causes CAS to persist, especially in head and neck cancer, where tumour location and its treatment interfere with nutritional interventions. Curcumin shows anti-inflammatory effects, including modulated CAS in animal and in vitro studies.

**Objective:**

The study aimed to determine the effect of curcumin to treat cancer anorexia-cachexia syndrome among current patients with locally advanced or advanced head and neck cancer.

**Methods:**

This constitutes a randomised, double-blind, placebo-controlled phase IIa study. Twenty patients with CAS in locally advanced or advanced head and neck cancer adequately nourished via a feeding tube were enrolled and randomised in a 1 : 1 ratio to receive oral curcumin (at a dose of 4000 mg daily) (*n* = 10) or placebo (*n* = 10) for 8 weeks. The primary endpoint was body composition (muscle mass, body fat mass, and basal metabolic rate). The secondary endpoints were handgrip muscle strength, body mass index, absolute lymphocyte count, and safety and toxicity.

**Result:**

There was a statistically significant benefit from curcumin on improving muscle mass compared with placebo (2.16% [95% confidence interval; CI, −0.75 to 5.07] vs. −3.82% [95% CI, −8.2 to 0.57]; *P*=0.019). The other parameters of body composition were not significant but tended to favour curcumin benefit. The body fat mass (−0.51 [95% CI, −21.89 to 20.86] vs. −8.97% [95% CI, −19.43 to 1.49]; *P*=0.432) and percentage of mean change in the basal metabolic rate were noted (BMR) (0.54% [95% CI, −1.6 to 2.67] vs. −1.61% [95% CI, −4.05 to 0.84]; *P*=0.153). Notably, patients treated with curcumin exhibited less reduction in handgrip muscle strength and absolute lymphocyte count but was not significant. Similarly, most adverse events were grade 1 in both groups.

**Conclusion:**

The curcumin add-on resulted in a significant increase in muscle mass than standard nutritional support. Furthermore, it may improve and delay a decrease in the other body composition parameters, handgrip strength, and absolute lymphocyte count. Curcumin was safe and well tolerated. This constitutes an unmet need for clinical trials. This trial is registered with NCT04208334.

## 1. Introduction

Cancer anorexia and cachexia syndrome (CAS) is defined as >5% weight loss in the previous six months or 2 to 5% weight loss with either a body mass index (BMI) of <20 kg/m^2^ or reduced muscle mass [1]. A high prevalence of CAS has been observed across malignancies, such as gastropancreatic cancer (80%), lung cancer/head and neck cancer (50%), and haematological malignancies (40%) [2–7]. CAS manifests various clinical syndromes, including anorexia, inflammation, insulin resistance, and increased muscle protein breakdown. These conditions are caused by various factors affecting metabolic pathways that lead to increases in morbidity and mortality among patients with advanced cancer. In addition, CAS is the major cause of death responsible for up to 20 to 30% among advanced stages of cancer [6].

The guidelines include a comprehensive therapeutic approach managing CAS, consisting of nutritional interventions, pharmacologic interventions, and other interventions (such as exercise). However, the evidence of pharmacologic intervention remains inconclusive regarding the benefits of many other agents [8–10].

CAS is a hypermetabolic state, which presents as an accelerated loss of skeletal muscle in the direction of chronic inflammation. Many studies have demonstrated the mechanisms underlying the metabolic and body composition changes via a potentially essential role for cytokine activation and the targeting of which comes across as skeletal muscle gene product [5, 7, 11, 12]. Additionally, the increase of the basal metabolic rate (BMR) or resting energy expenditure (REE) will possibly contribute to energy deficits leading to muscle wasting [13, 14].

Evidence supporting the hypothesis that TNF-alpha, IL-1 beta, and IL-6 serve as mediators in CAS comes from animal models and clinical studies [15]. Furthermore, the cytokines and mediators interact with their receptors on skeletal muscles and activate nuclear factor-kappa B (NF-kB). Nuclear factor-kappa B (NF-kB) is a REL family of a transcription factor, for which a major role in NF-kB transcription factor activation is skeletal muscle cell atrophy as a consequence of muscle protein degradation. Srivastava and Dhaulakhandi demonstrated that the decreasing level of the NF-kB light-chain was associated with decreased myolysis progression [16]. Therefore, inhibiting the NF-kB signalling pathway could be the rationale for the ameliorating process of cancer-induced muscle wasting.

Curcuminoids are the extracts from curcumin, which both in vitro and in vivo studies have shown the inhibitory effect of NF-kB through intracellular phosphorylation. Siddiqui et al. demonstrated that 100 mg/kg of curcumin prevented weight loss in MAC16 colon tumour mice. Moreover, the increasing dose of curcuminoid (up to 250 mg/kg) showed a 25% increase in body weight in mice [17]. Additionally, Gil da Costa et al. discovered preventing myolysis in HPV-16-infected mice from curcuminoids by the mechanism of downregulation of NF-kB synthesis resulted in increased muscle mass or delayed muscle wasting compared with controlled mice [18]. For curcumin in clinical studies, Gupta et al. showed that patients with solid cancer receiving chemotherapy and an additional 180 mg of curcumin for eight weeks showed a significant improvement in their quality of life and a declined NF-kB level compared with the controlled arm [19]. Recently, our previous study showed that 800 mg/day of curcumin delayed the progression of handgrip muscle strength loss and basal metabolic rate significantly among patients with solid cancer, as well as there were no serious adverse events [20]. This study aimed to determine the effect of up to 4,000 mg/day of curcumin on CAS in specific patients with locally advanced/advanced head and neck cancer stages. This randomised study was conducted in the unmet need area, where performing trials can be difficult.

## 2. Patients and Methods

### 2.1. Patient Selection

The eligibility criteria were as follows: (1) patients aged 18–75 years old; (2) diagnosis of locally advanced/advanced stage of head and neck cancer, including nasopharyngeal cancer; (3) patients receiving treatment with chemotherapy and/or radiation; (4) patients having at least 5% weight loss in the previous six months or 2 to 5% weight loss with a BMI of <20 kg/m^2^; (5) patients on a feeding tube, either nasogastric tube or gastrostomy tube; (6) ECOG PS was 0 to 2; and (7) patients having normal organ functions, for which the preserved hepatic function was defined as serum total bilirubin <2.0 mg/dL and serum aspartate aminotransferase (AST) and serum alanine aminotransferase (ALT) <3 times the upper-normal limit. However, patients with Gilbert's disease and liver metastasis maintained serum total bilirubin <3.5 mg/dL and serum aspartate aminotransferase (AST) and serum alanine aminotransferase (ALT) <5 times the upper-normal limit. Adequate renal function was defined as serum creatinine <2.0 mg/dL, and adequate bone marrow function was defined as absolute neutrophil count ≥1,500 cells/mm^3^, platelet count >100,000 cells/mm^3^, and haemoglobin ≥9 g/dL.

The exclusion criteria were as follows: (1) patients allergic to curcumin or its active components; (2) pregnancy or lactation; (3) indigestion or malabsorption; (4) biliary tract obstruction or biliary stone; (5) bleeding disorders; (6) patients on antiplatelets or anticoagulants; and (7) patients receiving any pharmacologic agents for appetite stimulation, such as progesterone analogue (megestrol acetate or medroxyprogesterone acetate). Furthermore, enrolled patients developing serious adverse events (such as anaphylaxis, Stevens–Johnson syndrome (SJS), toxic epidermal necrosis (TEN), or curcumin-induced hepatitis or acute kidney injury) during the course of the study were withdrawn from the study and treated at the discretion of the treating physician.

The study protocol was approved by the Institutional Review Boards, Royal Thai Army Medical Department. All patients provided written informed consent for study participation. This study was registered under the National Clinical Trial (NCT), number 04208334.

### 2.2. Sample Size Calculation

We calculated the sample size based on the comparable two-mean formula. This study showed 80% power at a two-tail 95% confidence interval to detect a 20% improvement in body composition (skeletal muscle). The sample size was eight patients on each arm, and treatment follow-up was assigned at four and eight weeks of study. A 20% loss follow-up rate was added to both arms, and the final sample size was ten patients in each group.

### 2.3. Dose Calculation

Curcuminoid dosage was calculated from the human equivalent dose (HED) on K14-HPV-16 mice dosage of 422.4 mg/kg in the Gil da Costa et al. study [18] and showed the benefits of delayed tumour progression, decreased inflammation and delayed myolysis based on dose translation from animal to human studies revisited formula [21]. HED involves 34.25 mg/kg, for which average dose is 1,600 mg/day based on a 50 kg adult. However, one study in Thailand using a dose of 1,600 mg/day of curcuminoids showed no significant body composition improvement. Nevertheless, a trend to delay handgrip muscle strength loss was noted in the curcuminoids group and absence of side effects [20]. The related studies showed no dose-limiting toxicity of curcuminoid up to 8,000 mg/day for eight weeks. Therefore, the dose was escalated to 4,000 mg/day to maximise the benefit of curcumin.

### 2.4. Curcumin and Matching Placebo Productions

Curcumin was manufactured under quality control by the Department of Pharmacology, Phramongkutklao Hospital and College of Medicine. Curcumin and matching placebo were in the form of 500 mg capsules. The curcuminoid content was at least 35 mg per 50 mg of curcumin, in total 500 mg of curcuminoid in each curcumin capsule, ascertained by high-performance liquid chromatography (HPLC). Matching placebo was made from probiotics and contained in a matched capsule. The manufacturing process for capsules met the requirement of Good Manufacturing Practice (GMP) and Good Laboratory Practice (GLP).

### 2.5. Study Design and Oversight

The CurChexia study comprised a double-blind, placebo-controlled, randomised, phase IIa trial comparing the efficacy of curcumin to treat cancer anorexia-cachexia syndrome compared with a matching placebo. Patients with locally/advanced stage of head and neck cancer underwent randomisation in a 1 : 1 ratio using the computer-generated block-of-four technique. Patients were stratified according to distant metastatic status (yes or no), type of cancer (head and neck squamous cell cancer vs. nasopharyngeal cancer), ECOG performance status (0 vs. 1 to 2), previous surgery status (yes or no), smoking history (yes or no), and comorbidity disease (diabetes, hypertension, or dyslipidemia). All investigators and patients are blinded from the selection and the prescription of drugs. The result of the body composition parameter was kept until the end of the study. The unblind protocol is used only for serious adverse events or at the end of the study.

Curcumin identical matching placebo was administered via a feeding tube at a dose of 2,000 mg twice daily in four capsules of 500 mg each for eight consecutive weeks. Laboratory values were monitored every two weeks in both groups. In addition, nutritionists assessed patients' nutritional status and calorie intake at every visit to ensure a calorie goal of 25 to 30 kcal/kg/day. The patients in both groups received standard polymeric formula for their enteral feeding for which caloric distribution comprised protein 15%, carbohydrate 56%, and fat 29%.

The study continued until the protocol was complete, except for unacceptable toxicity, patient's withdrawal from the study, or the principal investigator's decision to discontinue the study. All enrolled patients were not allowed to cross over to other cohorts.

All the authors had full access to all the data (including statistics reports and tables) in the study and were able to take responsibility for the integrity of the data and the accuracy of the data analysis.

### 2.6. Endpoints and Trial Assessments

The primary endpoint constituted changes in body composition, including lean body mass or muscle mass, body fat mass, and BMR. The body compositions were measured by the bioelectrical impedance assessment (BIA), which was analysed using a high-accuracy electromagnetic emission and measured in different kinds of tissues [22]. The patients underwent BIA at baseline, four weeks, and eight weeks at the end of the study. All BIA results were interpreted and reviewed by nutritionists and dieticians. The secondary endpoints were handgrip muscle strength, BMI, and absolute lymphocyte count level. The change in handgrip muscle strength was measured with a handheld dynamometer by one nutritionist. The standard BMI formula was used to calculate BMI change. The absolute lymphocyte count level change, reflecting the body's immune system, was measured using a blood test. Safety was assessed according to adverse events and clinically relevant changes in laboratory values. Adverse events were reported and recorded.

### 2.7. Statistical Analysis

We determine that the total 20 events of BIA assessment would provide the trial 90% power (at a two-sided alpha level of 5%) to show any significant difference in changes in body composition, muscle mass, body fat mass, and basal metabolic rate between the curcumin and the placebo-matched groups.

The baseline characteristics were analysed using descriptive statistics with statistical significance determined at *p* value <0.05.

The efficacy analysis for the primary endpoint and secondary endpoints was performed in the intention-to-treat population. Within-group means were determined using different comparison tests, and the independent *t*-test was used for between-group means. The outcomes reported analyses in overall mean change from baseline. We used the paired *t*-test to analyse mean different comparison tests. The chi-square test was used to determine the difference of adverse effects between the curcumin and placebo groups.

## 3. Results

### 3.1. Patients

**Figure 1:**
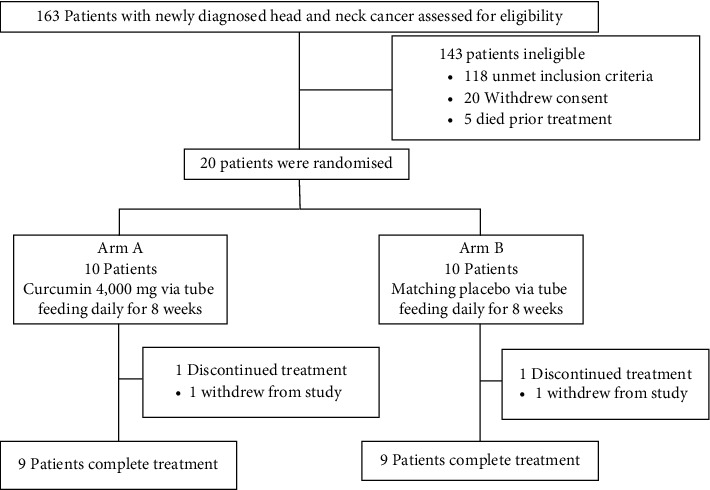
Consort flow diagram showing patients included in the study.

Between March 2020 and March 2021, patients underwent randomisation at Phramongkutklao Hospital, Bangkok, Thailand. A total of 20 patients were included in the intention-to-treat population. Of these patients, ten were assigned to receive curcumin, and another ten to receive matching placebo. Those enrolled patients had relatively good compliance with either curcumin or matching placebo. However, one patient in each group of the study withdrew from the study due to feeding tube obstruction at seven weeks after randomisation (Figure 1). Baseline characteristics of the patients are shown in Table 1. The data cut-off date was 31 March 2021.

### 3.2. Efficacy

#### 3.2.1. Primary Endpoint: Change in Body Composition

After eight consecutive weeks of treatments, we measured the body composition and handgrip strength using a BIA machine (InBody®).

*Muscle Mass*. Patients treated with curcumin had a change in muscle mass with a mean muscle mass gain of 0.46 (−0.2 to 1.12) kg after receiving curcumin for eight weeks, for which the mean muscle mass at week 0 was 24.04 ± 3.08 kg and at week 8 was 24.5 ± 2.72 kg. Moreover, patients treated with matching placebo had a change in muscle mass with a mean muscle loss of 1.05 (−2.34 to 0.24) kg after receiving matching placebo for eight weeks, for which the mean muscle mass at week 0 was 24.31 ± 5.56 kg and at week 8 was 23.26 ± 4.85 kg. The mean change in muscle mass among patients treated with curcumin significantly differed compared with patients treated with matching placebo (0.46 [−0.2 to 1.12] kg vs. −1.05 [−2.34 to 0.24] kg, *p* − value=0.03). In addition, the percent mean change of muscle mass at eight weeks among patients treated with curcumin significantly improved compared with patients treated with matching placebo (2.16%, 95% CI [−0.75 to 5.07] vs. −3.82%, 95% CI [8.2 to 0.57]; *p* − value=0.019).

**Figure 2:**
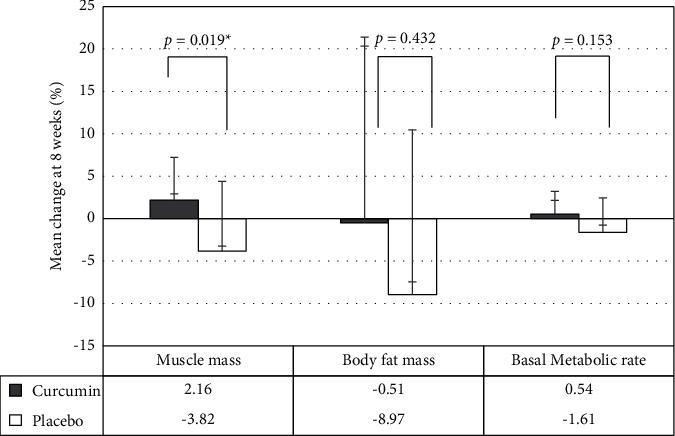
Comparison between the primary outcomes on the percentage of mean change of muscle mass, body fat mass, and basal metabolic rate between the curcumin and placebo groups. ^*∗*^statistically significant (*p* < 0.05).

*Body Fat Mass*. Patients treated with curcumin had changes in body fat mass with a mean body fat mass loss of 0.39 (−1.16 to 0.38) kg after receiving curcumin for eight weeks, where the mean body fat mass at week 0 was 8.64 ± 3.63 kg and at week 8 was 8.25 ± 3.47 kg. Patients treated with matching placebo also experienced changes in body fat mass with a mean body fat mass loss of 0.98 (−2.34 to 0.24) kg, where the mean body fat mass at week 0 was 10.7 ± 2.75 kg and at week 8 was 9.72 ± 2.88 kg. However, the percent mean change in body fat mass did not significantly differ between the two groups, for which the percentage of body fat mass loss was 0.51 (−21.89 to 20.86) kg among patients treated with curcumin and 8.97 (−19.43 to 1.49) among patients treated with matching placebo, *p* − value=0.43 (Figure 2).

*Basal Metabolic Rate*. The mean change in basal metabolic rate did not significantly differ between the two groups, which was 5.8 (−23.49 to 35.09) kcal among patients treated with curcumin and −22.2 (−56.59 to 12.19) kcal in the matching placebo group (*p* − value=0.17). Similarly, the percent change in basal metabolic rate did not significantly differ between the two groups, which was 0.54% (−1.6 to 2.67) among patients treated with curcumin and −1.61% (−4.05 to 0.84) in the matching placebo group, *p* − value=0.15 (Table 2).

#### 3.2.2. Secondary Endpoints

*Handgrip Strength*. For secondary endpoints, patients treated with curcumin had greater handgrip strength than patients treated with matching placebo, for which the mean change of handgrip strength among patients treated with curcumin and those with matching placebo were 0.61 (−2.17 to 3.39) kg and −0.62 (−3.03 to 1.79), respectively. However, statistically significant differences were observed between the two groups (*p* − value=0.95). Additionally, no statistically significant difference was noted in the percent change in handgrip strength between the two groups, which were 2.73% (−9.62 to 15.07) among patients with treated curcumin and −0.82% (−10.16 to 8.52) in the matching placebo group (*p* − value=0.93) (Table 3).

*Body Mass Index (BMI)*. For bodyweight issues, no substantial increase was found in BMI in both groups. However, patients treated with curcumin had less weight loss than the matching placebo group, for which the mean change of BMI was −0.1 kg (−0.71 to 0.51) among patients treated with curcumin and −0.88 kg (−1.85 to 0.08) in the matching placebo group, but no statistically significant difference was found between the two groups (*p* − value=0.2).

**Figure 3:**
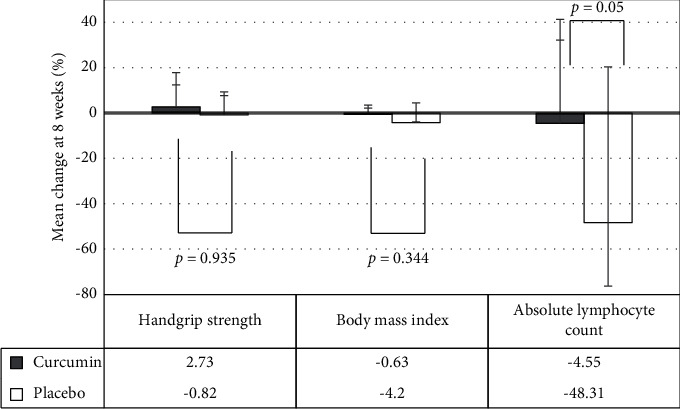
Comparison between the secondary outcomes on the percentage of mean change in handgrip strength, body mass index, and absolute lymphocyte count between the curcumin and placebo groups, ^*∗*^statistically significant (*p* < 0.05).

*Absolute Lymphocyte Count*. The reduction of absolute lymphocyte count among patients treated with curcumin was significantly lower than the matching placebo group, for which the mean change in absolute lymphocyte count was −227.7 cell/mm^3^ (−694.7 to 239.3) among patients treated with curcumin and −888.9 cell/mm^3^ (−1439.45 to −338.35) in the matching placebo group, *p* − value=0.05 (Figure 3).

### 3.3. Safety

A summary of adverse events is shown in Table 4. The common adverse events were nausea, diarrhoea, and headache in both groups. All adverse events were mild (grade I) symptoms in both groups. No dose reductions or dose discontinuation was observed in both groups. Nausea was more common among patients treated with curcumin than those in the matching placebo group, but without statistically significant difference. Patients in the matching placebo group developed more grade 1 diarrhoea than patients treated with curcumin, but without statistically significant difference between the two groups. No serious adverse events were noted in either group.

## 4. Discussion

The CurChexia study constituted a phase IIa (pilot), randomised, placebo-controlled study. We piloted among patients with locally advanced/advanced stage of head and neck cancer with CAS and receiving chemotherapy and/or radiation treatment. The prevalence of CAS among patients with locally advanced/advanced stage of head and neck cancer was high, up to 30 to 40%. Those patients often experience malnutrition either before or during treatment due to their disease severity and complications related to treatment with either radiation or chemotherapy. Therefore, the prophylactic feeding tube is efficient for ensuring adequate nutrition. For this reason, our study enrolled those particular patients with a feeding tube, either a gastrostomy tube or nasogastric tube, for easily tracking calorie intake, which may have reduced another confounding variable. However, a related study showed that adequate calorie intake alone could not be prevented from progressive CAS [23].

Regarding the pathogenesis of cancer cachexia, wasting of energy storage tissue of the skeletal muscle and body fat mass are potentially induced by cytokine activation and several tumour-derived substances, cachexia-inducing substances, and ubiquitin-proteasome pathways. Those cytokines and several substances directly interact with their receptors on skeletal muscle, leading to the activation of nuclear factor-kappa B (NF-kB). Inhibition of the NF-kB signalling pathway prevents cytokines and several cachexia-inducing substances to induce skeletal muscle loss. Therefore, NF-kB inhibition could be a pragmatic rationale to treat cancer cachexia. The underlying molecular mechanisms of curcumin prevent NF-kB activation by inhibiting phosphorylation and degradation of I*κ*B-alpha in the signalling pathway.

Using an animal model, a study was conducted on the effect of curcumin in Yoshida AH-130 ascites hepatoma mice receiving 20 mg/kg of curcuminoid for six days. The results showed the efficacy of curcuminoid delayed tumour growth. However, the muscle level and body weight were not increased [24]. On the other hand, the study of Gil da Costa et al. found that 422.4 mg/kg of curcumin improved tumour progression, decreased inflammation, and delayed myolysis regarding HPV-16-induced wasting syndrome in transgenic mice [21].

Presently, clinical studies exploring the efficacy of curcumin for treating cancer cachexia are lacking. Based on the study of Gil da Costa et al., an effective dose of curcumin was developed to improve cancer cachexia in HPV-16-induced wasting syndrome in a transgenic mice model. Using in vivo animal data to estimate the most biologically effective dose for curcumin in humans, the average dose determined was 1,700 mg/day based on a 50 kg adult. A related small clinical study suggested that, in patients with advanced solid cancer with cancer cachexia, administration of 1,600 mg/day of curcumin improved handgrip strength and basal metabolic rate. However, no statistically significant difference was found compared with the placebo (21). Therefore, our study escalated the dose of curcumin up to 4,000 mg/day due to its poor oral bioavailability [25], low absorption from the gut, and rapid metabolism. Additionally, other related studies showed no dose-limiting toxicity of curcuminoid at up to a dose of 8,000 mg/day for eight weeks [26, 27].

Our study investigated the efficacy of curcumin to improve CAS by using changes in body composition as the primary endpoint. It became clear that body weight was not a sufficient variable to evaluate response to treatment of cancer cachexia. Nevertheless, dual-energy X-ray absorptiometry (DEXA scan) or CT imaging techniques with novel software for skeletal muscle mass and body fat mass identification are ideal tools and potentially become the standard clinical practise [23]. However, body composition is the most reliable measure to determine the risk of cancer cachexia and skeletal muscle loss in the current practice. Notably, clinical studies revealed that the muscle quality or muscle strength mass ratio declines with ageing and significantly regresses over time [28].

Our study results showed significantly improved skeletal muscle mass with the use of curcumin compared with matching placebo, in which the difference in the percent change in muscle mass between the groups was significant. Moreover, body fat mass among patients treated with curcumin showed a slower decline than patients treated with matching placebo, even though no statistically significant difference was noted between the two groups, but the trend seems to favour curcumin. However, the basal metabolic rate among patients treated with curcumin at week 8 was marginally increased. While the basal metabolic rate among patients treated with matching placebo decreased, the difference in the percent change in the basal metabolic rate did not significantly differ between the two groups. In fact, the BMR is related to body weight using the formula BMR [cal/day] = 24 x bodyweight and can change over time and is affected by age, body weight, and height.

Moreover, body weight or BMI alone may not be adequate for assessing body composition. Therefore, the basal metabolic rate and body weight might not be a good candidate for assessing cancer cachexia. Furthermore, the international consensus group provides the definition of cancer cachexia as an alteration of body composition that is markedly characterised by ongoing skeletal muscle loss with or without loss of body fat mass, leading to negative protein and energy balance [1]. Therefore, it strengthens using appropriate skeletal muscle mass with or without body fat mass as surrogate endpoints to assess cancer cachexia.

Incorporating functional and physical measurements can assess cancer cachexia, such as handgrip strength or six-minute walk, yet they remain unclear whether they are reliable tools in practice because it depends on the patient's performance and severity of disease at that time. However, some clinical studies have demonstrated a positive correlation between muscle mass and muscle strength [29]. Our study showed that patients treated with curcumin improved handgrip strength at week 8, but loss of handgrip strength was observed in the matching placebo group. Furthermore, in vivo studies demonstrated curcumin's crucial significant immunomodulatory effect by stimulating PBMC proliferation and cytokine production [30]. Our study results supported the related in vivo study that curcumin administration for eight weeks resulted in less lymphocyte suppression compared with the matching placebo group. The potential role of curcumin regarding the immunomodulatory effect should be further investigated among patients with cancer during treatment with chemotherapy or immunosuppressive agents.

For safety endpoint, phase I studies suggested that curcumin at a dose up to 8,000 mg once daily orally can be safely administered without dose-limiting toxicity [26, 27]. Our study results demonstrated that curcumin was well tolerated at a dose of 4,000 mg/day and showed only mild grade of adverse events. No serious adverse events were reported.

The limitations of our study were as follows: first, this pilot study employed a relatively small sample size, leading to type II error skewing the results, decreasing the study's statistical power, and inflating false discovery rates; second, self-selection bias may have occurred because patients with locally advanced/advanced head and neck cancer with cancer cachexia tended to prefer receiving a specific intervention; third, the passage of time from receiving an adequate calorie intake via tube feeding influenced the dependent variable that could threaten the internal validity. And concern was evident that the use of probiotics as the placebo would interfere with the curcumin effects. However, at this time, no sufficient data show the benefit of probiotics to treat the CAS; fourth, this study enrolled particular patients with locally advanced/advanced head and neck cancer with cancer cachexia who were on a feeding tube that might threaten the external validity. Therefore, the results could not be generalised to all patients with cancer; and fifth, the anorexia symptom was not addressed and could not be interpreted because our study enrolled only those patients who were on tube feeding that may have obscured the symptom.

## 5. Conclusions

The pharmacologic intervention of cancer cachexia remains an unmet medical need. The pathogenesis of cancer cachexia is focused on underlying an alteration of body composition, in which the main component is loss of skeletal muscle mass. Nuclear factor-kappa B (NF-kB) is an important transcription factor for muscle protein degradation. Curcumin demonstrated the mechanism of inhibiting the NF-kB signalling pathway so that cancer-induced skeletal muscle loss could be ameliorated by curcumin. Our previous study showed a marginal benefit from treating cancer cachexia with an increase of 1,600 mg of curcumin.

Regarding its poor bioavailability, our phase IIa (pilot) study demonstrated the clinical benefits of body composition, including improved skeletal muscle mass and decreased loss of body fat mass with an increasing dose of curcumin up to 4,000 mg daily. Furthermore, improved handgrip strength and immunomodulatory effect appeared to favour the treatment with curcumin. Curcumin was safe and well tolerated up to the dose of 4,000 mg daily for eight weeks. Further larger phase IIb and phase III studies are required to prove the clinical benefits of curcumin to treat cancer cachexia.

## Figures and Tables

**Table 1:** Baseline characteristics of the patients (intention-to-treat population).

Characteristic	Curcumin group (*N* = 10)	Matching placebo group (*N* = 10)
Age (years)		
Median	58	60
Range	22–85	38–85
ECOG performance status score^†^, *n* (%)		
0	1 (10)	1 (10)
1	8 (80)	9 (90)
2	1 (10)	0 (0)
Head and neck cancer stage, no. (%)		
Locally advanced	8 (80)	9 (90)
Metastatic or recurrent	2 (20)	1 (10)
Head and neck cancer subgroup, no. (%)		
Nasopharyngeal cancer	1 (10)	1 (10)
Squamous cell head and neck cancer	9 (90)	9 (90)
Comorbid disease^‡^, *n* (%)	2 (20)	1 (10)
Surgery, *n* (%)	3 (30)	2 (20)
Smoking, *n* (%)	7 (70)	8 (80)
Treatment		
Concurrent chemoradiation, *n* (%)	6 (60)	8 (80)
Sequential chemoradiation, *n* (%)	2 (20)	0 (0)
Radiation only, *n* (%)	0 (0)	1 (10)
Palliative chemotherapy, *n* (%)	2 (20)	1 (10)
Mean daily calories intake ± SD (kcal/kg/day)	27.5 ± 2.500	27.92 ± 2.465

^†^Eastern Cooperative Oncology Group (ECOG) performance status scores range from 0 to 5, with 0 indicating no symptoms, 1 indicating mild symptoms, and a higher number indicating increasing degrees of disability. ^‡^Comorbidity disease defined by metabolic diseases, diabetes, hypertension, or dyslipidemia.

**Table 2:** Primary outcomes: muscle mass, body fat mass, and basal metabolic rate among the curcumin and placebo group patients, as an average change and percentage of an average change.

Group	Muscle mass, kg (mean ± SD)				Body fat mass, kg (mean ± SD)				Basal metabolic rate, kcal (mean ± SD)			
	Week 0	Week 8	Mean change (95% CI)	% Change (95% CI)	Week 0	Week 8	Mean change (95% CI)	% Change (95% CI)	Week 0	Week 8	Mean Change (95% CI)	% Change (95% CI)
Curcumin	24.04 ± 3.08	24.5 ± 2.72	0.46 (−0.2, 1.12)	2.16 (−0.75, 5.07)	8.64 ± 3.63	8.25 ± 3.47	−0.39 (−1.16, 0.38)	−0.51 (−21.89, 20.86)	1309.3 ± 89.92	1315.1 ± 77.9	5.8 (−23.49, 35.09)	0.54 (−1.6, 2.67)
Placebo	24.31 ± 5.56	23.26 ± 4.85	−1.05 (−2.34, 0.24)	−3.82 (−8.2, 0.57)	10.7 ± 2.75	9.72 ± 2.88	−0.98 (−2.08, 0.12)	−8.97 (−19.43, 1.49)	1339.8 ± 200.36	1317.6 ± 198.44	−22.2 (−56.59, 12.19)	−1.61 (−4.05, 0.84)
*p* values			0.03	0.019			0.334	0.432			0.178	0.153

**Table 3:** Secondary outcomes: handgrip strength, body mass index, and absolute lymphocyte count among the curcumin and placebo group patients, as an average change and percentage of an average change.

Group	Handgrip strength, kg (mean ± SD)				Body mass index (mean ± SD)				Absolute lymphocyte count, cell/mm^3^ (mean ± SD)			
	Week 0	Week 8	Mean change (95% CI)	% Change (95% CI)	Week 0	Week 8	Mean change (95% CI)	% Change (95% CI)	Week 0	Week 8	Mean change (95% CI)	% Change (95% CI)
Curcumin	26.24 ± 4.69	26.85 ± 6.18	0.61 (−2.17, 3.39)	2.73 (−9.62, 15.07)	18.49 ± 2.2	18.38 ± 2.47	−0.1 (−0.71, 0.51)	−0.63 (−4.1, 2.84)	1017.6 ± 693.4	789.9 ± 547.61	−227.7 (−694.7, 239.3)	−4.55 (−45.78, 36.69)
Placebo	26.19 ± 8.48	25.57 ± 8.24	−0.62 (−3.03, 1.79)	−0.82 (−10.16, 8.52)	19.53 ± 2.86	18.65 ± 2.47	−0.88 (−1.85, 0.08)	−4.2 (−8.64, 0.25)	1495 ± 756.88	606.1 ± 191.89	−888.9 (−1439.45, −338.35)	−48.31 (−68.62, −28)
*p* values			0.956	0.935			0.206	0.344			0.053	0.05

**Table 4:** Summary of adverse events.

Adverse event, *n* (%)	Curcumin group (*N* = 10)	Placebo-matched group (*N* = 10)	*p* values
	Grade I	Grade I	
Nausea	4 (40)	2 (20)	0.628
Diarrhoea	0 (0)	3 (30)	0.211
Headache	2 (20)	1 (10)	1.000
Hepatitis^#^	0 (0)	0 (0)	—
Acute kidney injury^#^	0 (0)	0 (0)	—

^#^Defined as a serious adverse event leading to the study's termination.

## Data Availability

All data generated or analysed during this study are included in this article. Further enquiries can be directed to the corresponding authors.
